# The effectiveness of a nationwide universal coverage campaign of insecticide-treated bed nets on childhood malaria in Malawi

**DOI:** 10.1186/s12936-016-1550-9

**Published:** 2016-10-18

**Authors:** Collins O. F. Zamawe, Kanan Nakamura, Akira Shibanuma, Masamine Jimba

**Affiliations:** Department of Community and Global Health, Graduate School of Medicine, The University of Tokyo, 7-3-1 Hongo, Bunkyo-ku, Tokyo, 113-0033 Japan

**Keywords:** Malaria, Insecticide-treated bed nets, Children, *Plasmodium falciparum*

## Abstract

**Background:**

Although the universal coverage campaign of insecticide-treated mosquito bed nets (ITNs) has been associated with improved malaria outcomes, recent reports indicate that the campaign is losing its sparkle in some countries. In Malawi, the universal coverage campaign was implemented in 2012, but its impacts are yet to be ascertained. Thus, this study examined the effects of the campaign on malaria morbidity among children in Malawi.

**Methods:**

This is a repeated cross-sectional study. The study used nationally-representative malaria indicator survey (MIS) data collected in 2012 and 2014. In total, the analysis included 4193 children between the ages of 6 and 59 months (2171 from 2012 MIS and 2022 from 2014 MIS). ITNs coverage and malaria morbidity before (2012 = pre-test/control) and after (2014 = post-test/treated) the universal coverage campaign of ITNs were compared. The treated and control samples were matched on measured relevant covariates using propensity scores.

**Results:**

The mean number of ITNs per household improved significantly from 1.1 (SD 1.0) in 2012 to 1.4 (SD 1.1) in 2014 (p < 0.001). Nonetheless, the prevalence of malaria among children increased considerably from 27.7 % (2012) to 32.0 % (2014) (p = 0.002). The risk of malaria was also significantly higher in 2014 compared to 2012 (RR = 1.14; 95 % CI 1.01–1.29). Besides, the use of bed nets was not significantly associated with malaria morbidity in 2014 (RR = 0.92; 95 % CI 0.76–1.12), but it was in 2012 (RR = 0.83; 95 % CI 0.70–1.00).

**Conclusions:**

The universal coverage campaign of ITNs was not associated with a reduced burden of malaria among children in Malawi. This was likely due to increased insecticide resistance, inconsistent use of bed nets and under-utilization of other methods of malaria control. This calls for a multifaceted approach in the fight against malaria instead of simple dependence on ITNs. In particular, local or community level malaria interventions should go hand in hand with the universal coverage campaign.

**Electronic supplementary material:**

The online version of this article (doi:10.1186/s12936-016-1550-9) contains supplementary material, which is available to authorized users.

## Background

The global incidence and mortality rate of malaria have fallen by 37 and 58 %, respectively in the last decade [1]. Nevertheless, malaria is still a major life-threatening disease in the world. For instance, almost half of the world’s population (3.3 billion) remains at risk of being infected with malaria and over 200 million cases of malaria and 438,000 malaria attributable deaths were reported in 2015 [2]. Globally, children aged under-five years are the most susceptible group to malaria [1, 2]. Malaria kills a child every minute and accounts for one-sixth of all childhood deaths in high malaria transmission areas [3]. In 2013, around 80 % of the malaria deaths occurred among under-five children [4].

The World Health Organization (WHO) recognizes five key interventions for preventing and treating malaria. These are prompt diagnosis and effective treatment of malaria, the use of insecticide-treated mosquito nets (ITNs) or long-lasting insecticidal nets (LLINs), indoor residual spraying, chemo-prevention and intermittent preventive therapy for pregnancy/infancy [5]. Since malaria is largely transmitted through mosquito bites, the use of ITNs is the most popular, practical and cost-effective intervention [6, 7]. Evidence is available to show that ITNs can save about six lives each year for every 1000 children [8].

The burden of malaria is heaviest in low income countries [2]. The recent world malaria report indicates that about 80 % of the global malaria cases and deaths in 2013 occurred in Africa, particularly in the sub-Saharan region [2]. Additionally, malaria is also one of the top ten causes of mortality in low income countries [2, 9]. Therefore, to ensure that all those at risk of malaria are protected (especially in malaria endemic countries), WHO recommended universal access to ITNs [10, 11].

Malawi is one of the malaria endemic countries. It is among the 18 high-risk countries accounting for 90 % of the estimated number of *Plasmodium falciparum* infections in sub-Saharan Africa [2]. Almost everyone in Malawi is at risk of malaria (>1 case/1000 population) [2]. In particular, about six million suspected malaria cases are treated each year and malaria is the number one cause of morbidity and mortality in the country [12]. Of the suspected malaria cases, about 50 % occur among under-five children [12]. Malaria is also responsible for about 40 % of the under-five children hospitalization annually [12, 13].

An ITN policy has been at the centre of malaria control in Malawi since 2006 [13]. In the first few years, the focus was on free distribution of ITNs to all under-five children and pregnant women during their first visit to a health facility/antenatal care [13, 14]. This approach proved insufficient to achieve the universal coverage of ITNs [14]. For that reason, a nationwide mass distribution of ITNs (hereinafter universal coverage campaign) was implemented in 2012 [12]. The goal was to have 90 % of all households owning at least one ITN and achieve a net utilization rate of 80 % [12]. About 5.6 million ITNs were distributed across the country [12, 13]. The campaign reached 87 % of the people who were registered and the number of persons per net ranged from 1.6 to 2.4. Specific details of the campaign, including logistics in the field are described elsewhere [12].

Even though the universal coverage campaign is evidence driven and has been shown to reduce malaria-associated morbidity and mortality [8, 15, 16], recent reports suggest that the campaign is losing its sparkle in some countries. In Burkina Faso, for instance, childhood malaria increased after the campaign [17]. Similarly, the scale-up of ITN coverage was not associated with decreased incidence of malaria in Zambia and Mali [18, 19]. Considering that huge amount of resources are being channelled towards the universal coverage campaign [2, 13], it is critical to understand how increased access to ITNs relates to the population health. This would ensure efficient use of the limited resources in the fight against malaria. So far, the effect of the universal coverage campaign in Malawi has not been ascertained. Therefore, this study examined the effectiveness of the universal coverage campaign on malaria morbidity among under-five children in Malawi.

## Methods

### Study design and data

This is a repeated cross-sectional study. Nationally representative data collected in 2012 and 2014 through the malaria indicator survey (MIS) were used. In 2012, the MIS was conducted from April to May [20]. This was just before the universal coverage campaign—though about 500,000 of the 5.6 million ITNs had already been distributed. The 2014 MIS was undertaken from May to June [21]. Thus, the 2012 and 2014 surveys were carried out around the same time of the year. In this study, bed net coverage and malaria morbidity among under five children before and after the universal coverage campaign were compared.

### Overview of the MIS: objectives, population and sampling

The MIS is a nationally representative cross-sectional study that is periodically conducted as part of the national malaria surveillance program in malaria burdened countries [20, 21]. In Malawi, the key objectives of MIS are to (a) monitor and evaluate the coverage and use of malaria control interventions, (b) assess knowledge, attitudes, and practices of malaria and (c) measure the prevalence of fever, malaria and anaemia among children [20, 21]. The survey is implemented by the Ministry of Health through the National Malaria Control Programme [20, 21].

In 2012 and 2014, MIS collected data from 3404 to 3405 households, respectively. These households were identified through a two-stage cluster sampling method. The first stage involved selecting 140 clusters (or enumeration areas) out of about 12,474 by means of probability proportional to size. In the second stage, 25 households were selected from each chosen cluster using a systematic random sampling approach. All women of reproductive age (15–49 years) and children aged between 6 and 59 months (hereinafter children) in the selected households were eligible to participate. For the children, malaria and haemoglobin tests were conducted in the field and a thick blood smear was taken for a confirmatory malaria parasite laboratory test.

### Data analysis and statistical procedures

MIS 2012 and 2014 children’s datasets were pooled. These datasets also contained information about the children’s mothers. In total, 4193 children were included in the analysis (2171 from 2012 MIS and 2022 from 2014 MIS). Variables were included in the analysis based on their relevance to the study objectives. The outcome variable was malaria status (positive or negative) based on confirmed laboratory results. Key covariates included ‘child slept under a bed net’ (‘yes’ if the child slept under bed net the night before the survey, otherwise ‘no’), number of bed nets per household and social-demographic characteristics of the child and his/her mother.

The analysis proceeded in three stages. First, descriptive statistics were computed and the predictors of malaria in 2012 and 2014 were separately examined. In particular, Chi-squared tests for independence were performed and multivariable logistic regression models were fitted. The level of significance was 5 % and we used 95 % confidence interval (CI). In each year (2014/2012), the urban–rural ratio of the selected clusters was not proportional to the population distribution in Malawi. For that reason, sample weights were used to make the data representative of the entire population [20, 21].

The second stage involved examining the impact of ITN scale-up on childhood malaria. The universal coverage campaign was the treatment variable or intervention. This study compared malaria morbidity before (2012 = control/pre-test) and after (2014 = treatment/post-test) the intervention. The 2012 and 2014 samples were matched using propensity scores to correct for selection bias [22]. The treated (2014) were matched with the control (2012) on the following covariates using a caliper of 0.001 [23]: age of the child, sex of the child, area of residence, cluster attitude, wealth index score, sex of the household head, years of education (mother), number of under five children (household), number of household members, literacy (mother) and mother’ access to malaria messages. Standardized differences (0.10 cut-off point) were used to evaluate how well the treated and control groups were balanced in the matched samples [23, 24]. Propensity score matching (PSM) minimized systematic differences between samples to acceptable levels (see Additional file 1). In addition to estimating the overall treatment effect, the samples were also stratified into rural and urban (area of residence).

Lastly, this study assessed the effect of ITNs on malaria morbidity. The use of bed net was the treatment variable or intervention. Malaria morbidity was compared between children who slept under a bed net (treated) and those who did not (control) after correcting for selection bias through PSM [22]. In 2014, data concerning the use of bed nets were available for 1934 out of 2022 participants. Of these, 1411 (73.0 %) used bed nets (treated) and 523 (27.0 %) did not (control). In 2012, the sample consisted of 2171 participants. However, data regarding the use of bed nets were available for 2011 participants, of whom 1238 (61.6 %) slept under a bed net (treated) and 773 (38.4 %) did not (control). The participants for each year were matched separately (see Additional file 2) using the same specifications as in stage two above. All analyses were performed using Stata 13.1 (StataCorp LP, Texas, USA).

### Ethics statement

The MIS data and permission to use it were obtained from the demographic health survey (DHS) program. Besides, the study protocol was reviewed and approved by the Research Ethics Committee of the University of Tokyo. The original study obtained ethical clearance from the Malawi’s National Health Sciences Research Committee (NHSRC). Mothers provided verbal informed consents for their children’s haemoglobin and malaria tests [20, 21].

## Results

**Fig. 1:**
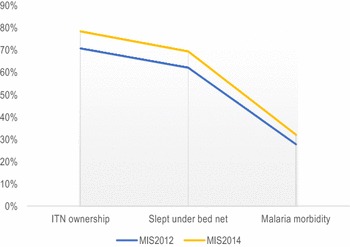
2012 and 2014 trends in ITN coverage and childhood malaria in Malawi (n = 4495—weighted). *MIS* malaria indicator survey, *ITN* insecticide treated bed net

The coverage and use of ITNs increased between 2012 and 2014 in Malawi. More precisely, the mean number of ITNs per household improved significantly from 1.1 (SD 1.0) in 2012 to 1.4 (SD 1.1) in 2014 (p < 0.001). The proportion of children who had at least one mosquito net also increased from 70.8 to 78.4 % during the same period (p < 0.001). Similarly, the proportion of children who slept under a bed net increased from 62.1 % (2012) to 69.4 % (2014) (p < 0.001). Notwithstanding, the national prevalence of malaria parasite among children increased considerably from 27.7 % (2012) to 32.0 % (2014) (p = 0.002). Figure 1 depicts ITN coverage, bed net use and malaria morbidity in 2012 and 2014. Social and demographic particulars of the participants in 2012 and 2014 with respect to malaria outcomes are described in Table 1.

**Table 1:** Under five children malaria morbidity in 2012 and 2014 by socio demographic characteristics (weighted, unmatched)

Variable name	Category	Year = 2012 (n = 2312)				Year = 2014 (n = 2183)			
		Malaria test outcome				Malaria test outcome			
		Total	Negative	Positive	p value	Total	Negative	Positive	p value
Age of the child (months)	Mean (SD)	31.4 (15.3)	30.5 (15.2)	34.5 (15.1)	<0.001*	30.9 (15.3)	30.0 (15.5)	35.1 (14.9)	<0.001*
Sex of the child	Female	1001 (52.9 %)	769 (53.0 %)	300 (54.0 %)	0.955**	1111 (50.9 %)	665 (51.5 %)	289 (47.4 %)	0.149**
	Male	892 (47.1 %)	681 (47.0 %)	255 (46.0 %)		1072 (49.1 %)	626 (48.5 %)	319 (52.6 %)	
Area of residence	Rural	2009 (86.9 %)	1211 (83.5 %)	527 (95.0 %)	<0.001*	1862 (85.3 %)	1052 (81.5 %)	582 (95.8 %)	<0.001*
	Urban	303 (13.1 %)	239 (16.5 %)	28 (5.0 %)		321 (14.7 %)	239 (18.5 %)	25 (4.2 %)	
Cluster attitude (KM)	Mean (SD)	0.9 (0.3)	0.9 (0.3)	0.9 (0.3)	0.169**	0.9 (0.3)	1.0 (0.0)	0.9 (0.0)	0.001*
Child slept under bed net	No	810 (37.9 %)	492 (36.6 %)	228 (44.2 %)	<0.001*	637 (30.6 %)	353 (28.3 %)	224 (39.2 %)	0.016*
	Yes	1327 (62.1 %)	852 (63.4 %)	288 (55.8 %)		1444 (69.4 %)	891 (71.7 %)	347 (60.8 %)	
Sex of household head	Female	497 (21.5 %)	319 (22.0 %)	115 (20.7 %)	0.632**	320 (14.7 %)	194 (15.0 %)	93 (15.4 %)	0.398**
	Male	1815 (78.5 %)	1130 (78.0 %)	440 (79.3 %)		1863 (85.3 %)	1098 (85.0 %)	513 (84.6 %)	
Age of the mother (years)	Mean (SD)	28.4 (6.7)	28.2 (6.5)	28.3 (6.9)	0.863**	28.0 (6.5)	28.1 (6.4)	28.4 (6.7)	0.411**
Wealth index score	Mean (SD)	2.8 (1.4)	3.3 (1.5)	2.5 (1.3)	<0.001*	2.8 (1.4)	3.3 (1.4)	2.5 (1.3)	<0.001*
Mother’s total years of education	Mean (SD)	4.8 (3.6)	5.8 (3.8)	3.9 (3.1)	<0.001*	5.4 (3.7)	6.3 (3.6)	4.7 (3.2)	<0.001*
Number of under five children (mother)	Mean (SD)	1.7 (0.7)	1.7 (0.7)	1.8 (0.7)	0.004*	1.7 (0.7)	1.6 (0.6)	1.7 (0.7)	0.002*
Number of bed nets (household)	Mean (SD)	1.1 (1.0)	1.2 (1.1)	0.9 (0.9)	<0.001*	1.4 (1.1)	1.6 (1.1)	1.4 (1.1)	0.001*
Child’s mother can read	No	846 (36.6 %)	474 (32.7 %)	262 (47.1 %)	<0.001*	682 (31.2 %)	354 (27.4 %)	253 (41.6 %)	<0.001*
	Yes	1466 (63.4 %)	976 (67.3 %)	293 (52.9 %)		1501 (68.8 %)	938 (72.6 %)	354 (58.4 %)	
Mother heard messages about malaria (<6 weeks ago)	No	1586 (73.4 %)	973 (70.6 %)	407 (81.9 %)	<0.001*	1593 (78.9 %)	938 (76.4 %)	455 (84.5 %)	0.203**
	Yes	574 (26.6 %)	405 (29.4 %)	90 (18.1 %)		427 (21.1 %)	290 (23.6 %)	83 (15.5 %)	
Mother knowns mosquito bites cause malaria	No	149 (6.9 %)	70 (5.1 %)	54 (10.8 %)	<0.001*	286 (14.2 %)	153 (12.5 %)	94 (17.5 %)	0.084**
	yes	2011 (93.1 %)	1307 (94.9 %)	443 (89.2 %)		1734 (85.8 %)	1074 (87.5 %)	444 (82.5 %)	
Mother knows fever is the main sign of malaria	No	190 (8.8 %)	127 (9.2 %)	32 (6.5 %)	0.082**	266 (13.2 %)	167 (13.6 %)	44 (8.1 %)	0.020*
	Yes	1970 (91.2 %)	1250 (90.8 %)	464 (93.5 %)		1754 (86.8 %)	1060 (86.4 %)	495 (91.9 %)	
Mother knowns sleeping under ITN prevents Malaria	No	1187 (54.9 %)	755 (54.8 %)	283 (57.0 %)	0.245**	1514 (74.9 %)	890 (72.5 %)	422 (78.5 %)	0.023*
	Yes	973 (45.1 %)	623 (45.2 %)	214 (43.0 %)		506 (25.0 %)	337 (27.5 %)	116 (21.5 %)	
Mother knows mosquito repellant prevents malaria	No	2099 (97.2 %)	1333 (96.8 %)	490 (98.6 %)	0.064**	1991 (98.5 %)	1202 (97.9 %)	537 (99.8 %)	0.017*
	Yes	61 (2.8 %)	11 (3.2 %)	7 (1.4 %)		29 (1.5 %)	26 (2.1 %)	1 (0.3 %)	
Mother knowns mosquito coil prevents malaria	No	2114 (97.9 %)	1343 (97.5 %)	489 (98.5 %)	0.112**	2003 (99.2 %)	1214 (98.9 %)	535 (99.4 %)	0.800**
	Yes	46 (2.1 %)	35 (2.5 %)	7 (1.5 %)		17 (0.8 %)	13 (1.1 %)	3 (0.6 %)	
Mother knowns cutting grass around house prevents malaria	No	1950 (90.3 %)	1238 (89.8 %)	455 (91.6 %)	0.046*	1905 (94.3 %)	1145 (93.3 %)	519 (96.4 %)	0.059**
	Yes	210 (9.7 %)	140 (10.2 %)	42 (8.4 %)		115 (5.7 %)	82 (6.7 %)	19 (3.6 %)	
Mother knowns children are vulnerable to Malaria	No	275 (12.7 %)	159 (11.6 %)	85 (17.1 %)	0.002*	388 (19.2 %)	232 (18.9 %)	102 (19.0 %)	0.864**
	Yes	1885 (87.3 %)	1218 (88.4 %)	411 (82.9 %)		1632 (80.8 %)	995 (88.1 %)	436 (81.0 %)	

*SD* standard deviation, *KM* kilometres, *ITN* insecticide-treated bed net

* Significant (p ≤ 0.05); ** non significant (p > 0.05)

Besides, the determinants of malaria morbidity among children were examined. In 2012, the odds of being infected with malaria were significantly lower among children who slept under a bed net than those who did not (AOR = 0. 65; 95 % CI 0.47–0.89). In contrast, sleeping under bed net was not a significant predictor of malaria in 2014 (AOR = 0.77; 95 % CI 0.57–1.06). In both years, the number of bed nets per household was not significantly associated with malaria morbidity among children. Table 2 presents the predictors of childhood malaria in 2012 and 2014, including all the variables that were included (adjusted for) in the models.

**Table 2:** Predictors of malaria morbidity in under five children (unmatched data)

Variable	Year 2012			Year 2014		
	AOR	95 % CI	p value	AOR	95 % CI	p value
Child slept under bed net	0.65	0.47–0.89	0.007*	0.77	0.57–1.06	0.109**
Child is male	0.92	0.73–1.15	0.463**	1.15	0.92–1.44	0.215**
Age of the child	1.02	1.01–1.03	<0.001*	1.02	1.02–1.03	<0.001*
Residential area is urban	0.42	0.26–0.67	<0.001*	0.35	0.21–0.59	<0.001*
Cluster altitude (KM)	1.17	0.83–1.66	0.380**	0.54	0.37–0.77	0.001*
Wealth index score	0.87	0.79–0.96	0.004*	0.80	0.73–0.88	<0.001*
Age of the mother	0.98	0.96–1.00	0.017*	0.99	0.97–1.01	0.342**
Mother’s years of education	0.93	0.88–0.99	0.023*	0.97	0.92–1.00	0.240**
Mother can read	1.22	0.83–1.79	0.325**	0.88	0.61–1.28	0.501**
Number of under five children	1.21	1.04–1.42	0.015*	1.25	1.05–1.48	0.011*
Number of bed nets (household)	1.14	0.98–1.33	0.102**	0.93	0.81–1.08	0.367**
Male household head	1.11	0.84–1.46	0.464**	0.96	0.71–1.29	0.768**
Heard malaria messages (<6 months ago)	0.66	0.49–0.87	0.004*	0.79	0.58–1.07	0.126**
Mother knowns mosquito bites cause malaria	0.49	0.32–0.74	0.001*	0.90	0.64–1.25	0.521**
Mother knows fever is the main sign of malaria	1.70	1.08–2.69	0.023*	1.85	1.26–2.71	0.002*
Mother knowns ITN prevents malaria	1.17	0.92–1.48	0.205**	1.00	0.76–1.32	0.999**
Mother knows mosquito repellant prevents malaria	0.58	0.24–1.40	0.227**	0.22	0.04–1.29	0.094**
Mother knowns mosquito coil prevents malaria	1.33	0.56–3.20	0.517**	0.60	0.16–2.20	0.437**
Mother knowns cutting grass around house prevents malaria	0.86	0.58–1.27	0.451**	0.89	0.51–1.56	0.693**
Mothers knowns children are vulnerable to malaria	0.71	0.51–0.99	0.043*	1.32	0.98–1.78	0.071**

*CI* confidence interval, *KM* kilometres, *ITN* insecticide-treated bed net

* Significant (p ≤ 0.05); ** non significant (p > 0.05)

Furthermore, the effect of ITN scale-up on childhood malaria in Malawi was examined using the matched data. Particularly, this study compared malaria morbidity in 2014 (treatment) and 2012 (control). Overall, the risk of malaria was 14 % higher in 2014 than 2012 (RR = 1.14; 95 % CI 1.01–1.29). Said differently, Malawian children were at a significantly greater risk of malaria after the universal coverage campaign than before (Table 3).

**Table 3:** The effects of the universal coverage campaign (UCC) of ITNs and the use of bed nets on childhood malaria in Malawi

Year/period	Intervention	Level of analysis	Malaria outcomes				RR (95 % CI)	p value
			Control (n)		Treatment (n)			
			negative	Positive	Negative	Positive		
2012 vs 2014	UCC of ITNs	Overall	1359	381	1205	401	1.14 (1.00–1.29)	0.036*
2012 vs 2014	UCC of ITNs	Rural	898	373	779	358	1.07 (0.95–1.21)	0.254**
2012 vs 2014	UCC of ITNs	Urban	454	59	373	37	0.78 (0.53–1.16)	0.223**
2014	Bed net use	Overall	313	115	682	224	0.92 (0.76–1.12)	0.399**
2014	Bed net use	Rural	227	100	448	197	1.00 (0.82–1.22)	0.990**
2014	Bed net use	Urban	96	5	110	7	1.21 (0.40–3.69)	0.740**
2012	Bed net use	Overall	465	164	719	200	0.83 (0.70–1.00)	0.049*
2012	Bed net use	Rural	304	140	451	168	0.86 (0.71–1.04)	0.119**
2012	Bed net use	Urban	156	29	121	11	0.53 (0.28–1.03)	0.060*

*RR* risk ratio, *ITNs* insecticide-treated bed nets, *CI* confidence interval

* Significant (p ≤ 0.05); ** non significant (p > 0.05)

In addition, this study also investigated the impact of using bed nets on malaria outcomes among children in 2012 and 2014. In 2014, the overall risk of malaria was lower among children who used bed nets compared to those who did not (RR = 0.92; 95 % CI 0.76–1.12). However, the result was not statistically significant, even after stratification by areas of residence (rural/urban). In contrast, the use of bed nets in 2012 was significantly associated with decreased risk of childhood malaria (RR = 0.83; 95 % CI 0.70–1.00). Detailed results are provided in Table 3.

## Discussion

There are three key findings from this study. First, the 2012 universal coverage campaign improved both bed net coverage and use among children in Malawi. Secondly, the use of bed nets was significantly associated with reduced risk of childhood malaria in 2012, but not in 2014. This simply suggests that ITNs were relatively ineffective after the 2012 universal coverage campaign in Malawi. Lastly, the prevalence of malaria among children in Malawi was higher in 2014 than 2012. In other words, the universal coverage campaign was not associated with improved malaria outcomes.

Improved access to ITNs between 2012 and 2014 was not associated with reduced malaria morbidity among children in Malawi. This finding challenges the current public health understanding that the universal coverage campaign reduces the burden of malaria [8, 25–27]. Until now, the effects of ITN scale-up have been uneven. For example, malaria morbidity/mortality declined in south-west Cameroon, rural Tanzania and Rwanda after the coverage of ITNs was enhanced [28–30]. In contrast, higher/unchanged malaria prevalence/mortality were reported in Zambia, Burkina Faso, Mali and western Myanmar following the ITN scale-up [17, 19, 31]. In some cases, better access to ITNs was accompanied by reduced incidence of malaria at the outset, but the gains could not be sustained [32, 33]. These variations could be because most of the studies were confined to a particular population or setting in their respective countries [31, 34]. This study analysed nationally representative data with a special focus on children. As such, the results provide a richer or wider perspective of the impact of the universal coverage campaign.

Besides, ITN usage had an inconsequential impact on malaria morbidity in Malawi, especially after the universal coverage campaign. As noted, the use of ITNs provided substantial protection against childhood malaria in 2012, but the effects attenuated and became insignificant in 2014. The rise of insecticide resistance among Anopheles mosquitoes is one of the plausible reasons for this phenomenon [35, 36]. This hypothesis concurs with the findings of a study in Malawi, which has recently reported that there is reduced efficacy of bed nets in the country as a consequence of increased intensity of insecticide resistance [37]. Related outcomes have also been reported by another study in Mozambique, which has clearly shown that resistance to pyrethroids is extremely high in the country [36]. Therefore, amid serious concerns of insecticide resistance, the universal coverage campaign may not effectively reduce the burden of malaria in affected countries.

Comparable studies have attributed the reduced efficacy of ITNs to the community-wide effects of ITNs [17, 28, 38]. The reasoning is that ITNs do not only protect individuals from direct mosquito bites, they also kill the mosquitoes and thereby protecting from malaria even those who do not sleep under bed nets [38]. Thus, increased ITN coverage reduces to a greater extent the swarm of mosquitoes in an area and in so doing protecting from malaria more and more people with no access to ITNs. This notion provides an alternative explanation about why malaria morbidity was not significantly different between users and non-users of bed nets after ITN scale-up in Malawi. Nonetheless, the community-wide effects of ITNs fall short of elucidating the impact of the universal coverage campaign in Malawi. In general, the campaign increased ITN coverage and this suggests relatively far-reaching community-wide effects. As a result, malaria prevalence was not supposed to increase in 2014.

Knowledge about the causes of malaria (i.e. mosquito bites can cause malaria) and malaria prevention/control strategies (i.e. sleeping under ITNs prevents malaria) decreased among the children’s mothers between 2012 and 2014 in Malawi. Existing literature point out that knowledge of malaria transmission and prevention is associated with regular use of ITNs [39–41]. Accordingly, inconsistent use of ITNs was bound to be greater in 2014. Since the success of the universal coverage campaign partly depends on stable use of ITNs by those at risk of malaria, noncompliance might have contributed to the increase in malaria morbidity among Malawian children in 2014.

Furthermore, awareness of interventions that may limit malaria transmission before sleep time (i.e. cutting grass around the house, mosquito repellent and mosquito coil) was lower among the children’s mothers in 2014 compared to 2012. Because of the limited protection provided by ITNs (usually during bed time only) [42], supplementary interventions play a crucial role in malaria control. In Kenya and Myanmar, for example, ITNs offered incomplete protection against malaria due to early biting habits of the vectors [31, 42]. Due to low awareness, it is likely that additional malaria control methods were relatively under-utilized in 2014 [43, 44]. This resonate calls for broader vector control strategies to limit malaria transmission before bedtime [42].

Taken together, insecticide resistance and inadequate knowledge of malaria transmission and prevention pose a serious threat to the efficacy of ITNs and the future of the universal coverage campaign. This highlights the need to constantly monitor the level of insecticide resistance and the effects of ITNs to ensure that all people at risk of malaria are protected. Moreover, there is an urgent need to intensify and regularly update resistance management strategies. In the long run, alternative insecticides should be identified or developed. An integrated approach is called for to malaria control in lieu of simple dependence on ITNs. In particular, effective local malaria control strategies (i.e. fill in puddles and clearing bushes around residential areas) should be preserved and promoted in malaria endemic areas. Accordingly, the universal coverage campaign should go hand in hand with intensive community based malaria awareness campaigns.

This study had limitations and strengths. First, both 2012 and 2014 MIS relied on reported data except for malaria status. So, recall and social desirability biases cannot be ruled out. The strengths of this study include the use of nationally representative data with relatively large sample sizes. Although each survey was cross-sectional in nature, the pooling of the two datasets permitted comparison of the study outcomes over time. Moreover, PSM allowed to balance measured covariates across 2012 and 2014 samples [24]. Thus, the study design mimicked some of the characteristics of a randomized controlled trial [23].

## Conclusions

The universal coverage campaign improved both the access and use of bed nets among children between 2012 and 2014 in Malawi. Nonetheless, the campaign was not associated with a reduced burden of malaria. This is likely due to increased insecticide resistance, inconsistent use of bed nets and under-utilization of other malaria control methods. These observations accentuate the need for a composite intervention in malaria control. Thus, while applauding the massive contribution of ITNs in the fight against malaria, it is important to recognize that a multifaceted approach is required to effectively reduce the burden of malaria. To this end, the utility of local solutions and community level approaches in malaria control should be stressed.

## Additional files

**Additional file 1.** Survey participants (children) after (2014) and before (2012) the universal coverage campaign in Malawi matched on relevant covariates.

**Additional file 2.** Users and non-users (children) of bed nets matched in Malawi matched on relevant covariates.

## Abbreviations

ITNs: insecticide-treated mosquito bed nets
LLINs: lost lasting insecticidal treated mosquito bed nets
SD: standard deviation
CI: confidence interval
PSM: propensity score matching
WHO: World Health Organization
MIS: malaria indicator survey
DHS: demographic health survey
AOR: adjusted odds ratio
RR: risk ratio
