# Supplementary material for: The effectiveness of a nationwide universal coverage campaign of insecticide-treated bed nets on childhood malaria in Malawi
Source: Malar J. 2016 Oct 18;15:505. doi: 10.1186/s12936-016-1550-9 (PMC5070233; doi:10.1186/s12936-016-1550-9)
Supplement: Supplementary file 1 — Additional file 1. Survey participants (children) after (2014) and before (2012) the universal coverage campaign in Malawi matched on relevant covariates. [file 12936_2016_1550_MOESM1_ESM.docx]

| **Supplemental item 1: Survey participants (children) after (2014) and before (2012) the universal coverage campaign in Malawi matched on relevant covariates** | | | | |
| --- | --- | --- | --- | --- |
| **Covariates** | **Category** | **2014 sample** | **2012 sample** | **SD (% bias)** |
| Age of the child | Unmatched (mean) | 31.3 | 31.5 | -1.3 |
|  | Matched (mean) | 31.3 | 31.5 | -1.5 |
| Male child | Unmatched (prop.) | 0.5 | 0.5 | 6.8 |
|  | Matched (prop.) | 0.5 | 0.5 | 3.4 |
| Urban resident | Unmatched (prop.) | 0.3 | 0.3 | 0.9 |
|  | Matched (prop.) | 0.3 | 0.3 | -2.7 |
| Cluster altitude (KM) | Unmatched (mean) | 0.9 | 0.9 | 7.6 |
|  | Matched (mean) | 0.9 | 0.9 | 3.8 |
| Wealth index score | Unmatched (mean) | 3.1 | 3.2 | -4.7 |
|  | Matched (mean) | 3.1 | 3.1 | -0.5 |
| Male household head | Unmatched (prop.) | 0.8 | 0.8 | 13.3 |
|  | Matched (prop.) | 0.8 | 0.8 | 1.9 |
| Mother's years of education | Unmatched (mean) | 6.0 | 5.5 | 14.6 |
|  | Matched (mean) | 6.0 | 6.1 | -3.0 |
| Mother can read | Unmatched (prop.) | 0.8 | 0.7 | 11.1 |
|  | Matched (prop.) | 0.8 | 0.8 | -1.8 |
| Mother heard malaria messages (<6months) | Unmatched (prop.) | 0.2 | 0.3 | -10.7 |
|  | Matched (prop.) | 0.2 | 0.2 | -0.9 |
| Number of under five children | Unmatched (mean) | 1.6 | 1.7 | -12.8 |
|  | Matched (mean) | 1.6 | 1.6 | 4.2 |
| Number of household members | Unmatched (mean) | 5.4 | 5.3 | 1.0 |
|  | Matched (mean) | 5.3 | 5.2 | 4.6 |
| **Total samples** | **Unmatched** | **1,638** | **1,784** | 32.8 |
|  | **Matched** | **1,606** | **1,740** | 0.9 |

SD: standard differences; Prop: proportion; KM: kilometres
