# Supplementary material for: The effectiveness of a nationwide universal coverage campaign of insecticide-treated bed nets on childhood malaria in Malawi
Source: Malar J. 2016 Oct 18;15:505. doi: 10.1186/s12936-016-1550-9 (PMC5070233; doi:10.1186/s12936-016-1550-9)
Supplement: Supplementary file 2 — Additional file 2. Users and non-users (children) of bed nets matched in Malawi matched on relevant covariates. [file 12936_2016_1550_MOESM2_ESM.docx]

| **Supplemental item 2: Users and non-users (children) of bed nets matched in Malawi matched on relevant covariates** | | | | | | | |
| --- | --- | --- | --- | --- | --- | --- | --- |
| **Variable name** | **Category** | **Bed net use in 2014** | | | **Bed net use in 2012** | | |
|  |  | **Yes (T)** | **No (C)** | **SD** | **Yes (T)** | **No (C)** | **SD** |
| Age of the child | Unmatched (mean) | 30.7 | 32.5 | -11.2 | 31.0 | 32.1 | -7.1 |
|  | Matched (mean) | 31.6 | 31.6 | -0.1 | 31.1 | 30.1 | 6.9 |
| Male child | Unmatched (prop.) | 0.5 | 0.5 | -6.4 | 0.5 | 0.5 | 2.3 |
|  | Matched (prop.) | 0.5 | 0.5 | 1.4 | 0.5 | 0.5 | -2.2 |
| Urban resident | Unmatched (prop.) | 0.3 | 0.2 | 17.3 | 0.3 | 0.3 | -1.9 |
|  | Matched (prop.) | 0.3 | 0.3 | 1.1 | 0.3 | 0.3 | -3.4 |
| Cluster altitude (KM) | Unmatched (mean) | 0.9 | 1.0 | -7.0 | 0.9 | 0.9 | -18 |
|  | Matched (mean) | 0.9 | 0.9 | 2.4 | 0.9 | 0.9 | 0.3 |
| Wealth index score | Unmatched (mean) | 3.2 | 2.8 | 29.5 | 3.3 | 3.0 | 19.7 |
|  | Matched (mean) | 3.0 | 3.0 | 0.1 | 3.2 | 3.2 | -1.8 |
| Male household head | Unmatched (prop.) | 0.9 | 0.8 | 9.7 | 0.8 | 0.8 | 8.7 |
|  | Matched (prop.) | 0.8 | 0.8 | -1.3 | 0.8 | 0.8 | 4.9 |
| Mother's years of education | Unmatched (mean) | 6.5 | 5.0 | 42.2 | 6.0 | 4.9 | 29.3 |
|  | Matched (mean) | 5.9 | 5.8 | 3.0 | 5.6 | 5.7 | -2.1 |
| Mother can read | Unmatched (prop.) | 0.8 | 0.7 | 23.4 | 0.7 | 0.7 | 16.3 |
|  | Matched (prop.) | 0.8 | 0.7 | 3.3 | 0.7 | 0.7 | 0.5 |
| Mother heard malaria messages (<6months) | Unmatched (prop.) | 0.3 | 0.2 | 22.3 | 0.3 | 0.2 | 11.2 |
|  | Matched (prop.) | 0.2 | 0.2 | -2.6 | 0.3 | 0.3 | 3.4 |
| Number of under five children | Unmatched (mean) | 1.6 | 1.6 | -4.3 | 1.6 | 1.7 | -2.7 |
|  | Matched (mean) | 1.6 | 1.6 | -0.5 | 1.7 | 1.7 | 3.6 |
| Number of household members | Unmatched (mean) | 5.3 | 5.5 | -10.3 | 5.2 | 5.3 | -9.2 |
|  | Matched (mean) | 5.3 | 5.3 | -0.1 | 5.2 | 5.2 | -0.8 |
| **Total samples** | **Unmatched** | **1,151** | **428** | **51.7** | **1,029** | **629** | **46.8** |
|  | **Matched** | **906** | **428** | **5.5** | **919** | **629** | **11.6** |

SD: Standardised differences (% bias); Prop: proportion; T: Treatment; C: control; KM: Kilometres
